# Supplementary material for: Discovery of a potent anti-Zika virus benzamide series targeting the viral protein NS4B
Source: PLoS Pathog. 2026 Apr 3;22(4):e1013609. doi: 10.1371/journal.ppat.1013609 (PMC13065080; doi:10.1371/journal.ppat.1013609)
Supplement: S1 Fig — (DOCX) [file ppat.1013609.s001.docx]

S1 Fig. Effect of supplementary uridine on antiviral effect.


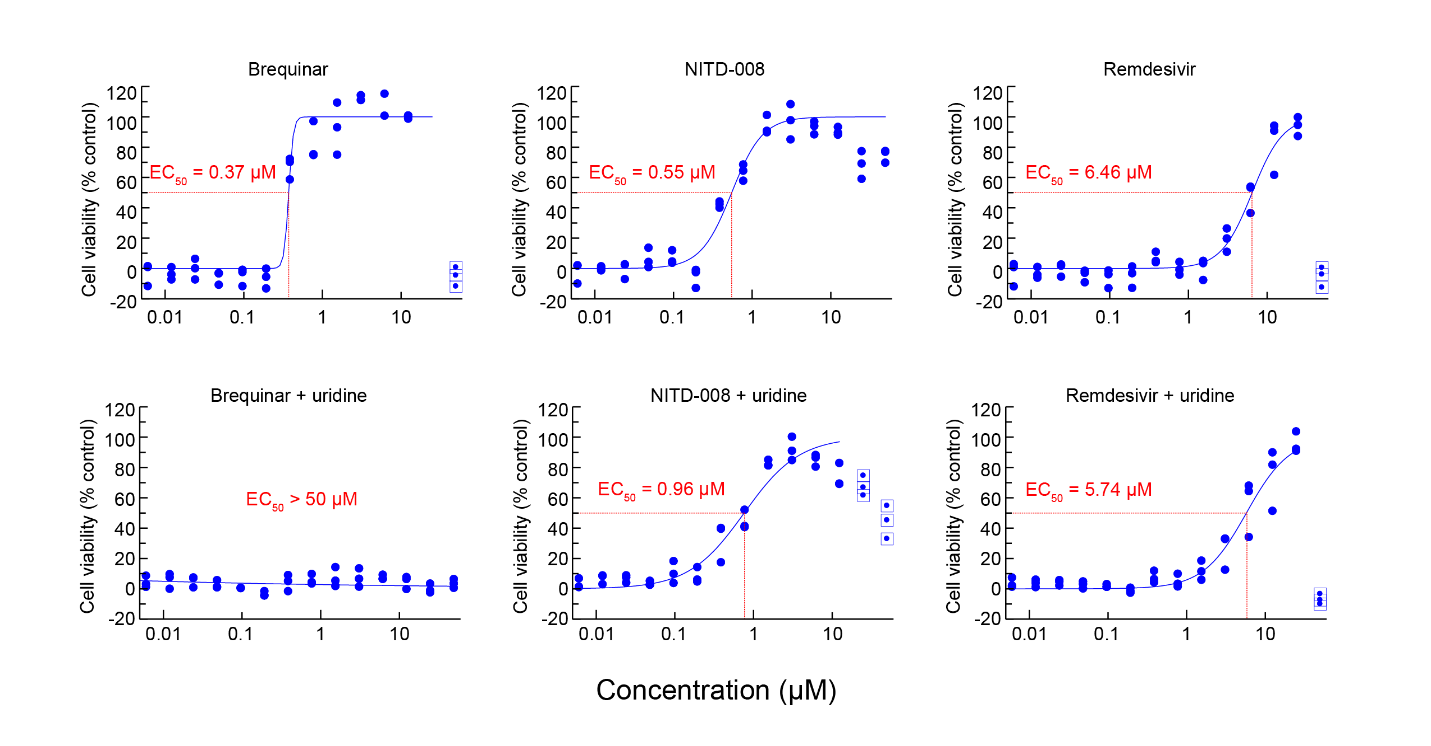


Vero 76 cells were infected with ZIKV (strain PL Cal) at MOI of 0.05 in the presence or absence of supplementary uridine (50 µM) in the media. Cell viability protected by the treatment from the virus-induced CPE was measured by using CellTiter-Glo as described in the Methods and Materials.
