# Supplementary figures and images for: Discovery of a potent anti-Zika virus benzamide series targeting the viral protein NS4B
Source: PLoS Pathog. 2026 Apr 3;22(4):e1013609. doi: 10.1371/journal.ppat.1013609 (PMC13065080; doi:10.1371/journal.ppat.1013609)

Fig. S2. A flow diagram of various assays used in the HTS of 650K compounds and follow-up assays.


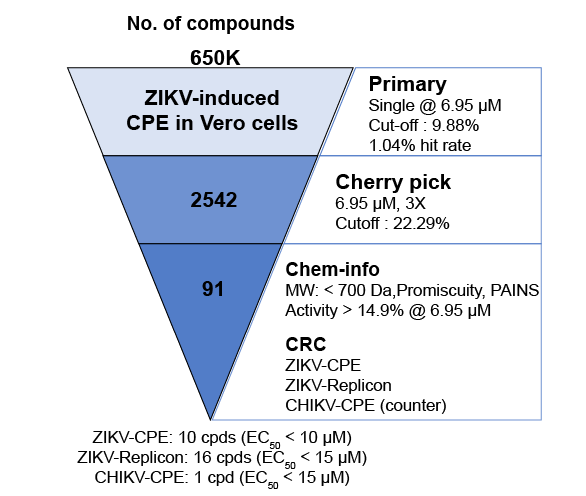

Supplement: S2 Fig — (DOCX) [file ppat.1013609.s002.docx]
