# Supplementary material for: Discovery of a potent anti-Zika virus benzamide series targeting the viral protein NS4B
Source: PLoS Pathog. 2026 Apr 3;22(4):e1013609. doi: 10.1371/journal.ppat.1013609 (PMC13065080; doi:10.1371/journal.ppat.1013609)
Supplement: S3 Fig — (DOCX) [file ppat.1013609.s003.docx]

S3 Fig. Time course of viral protein expression.


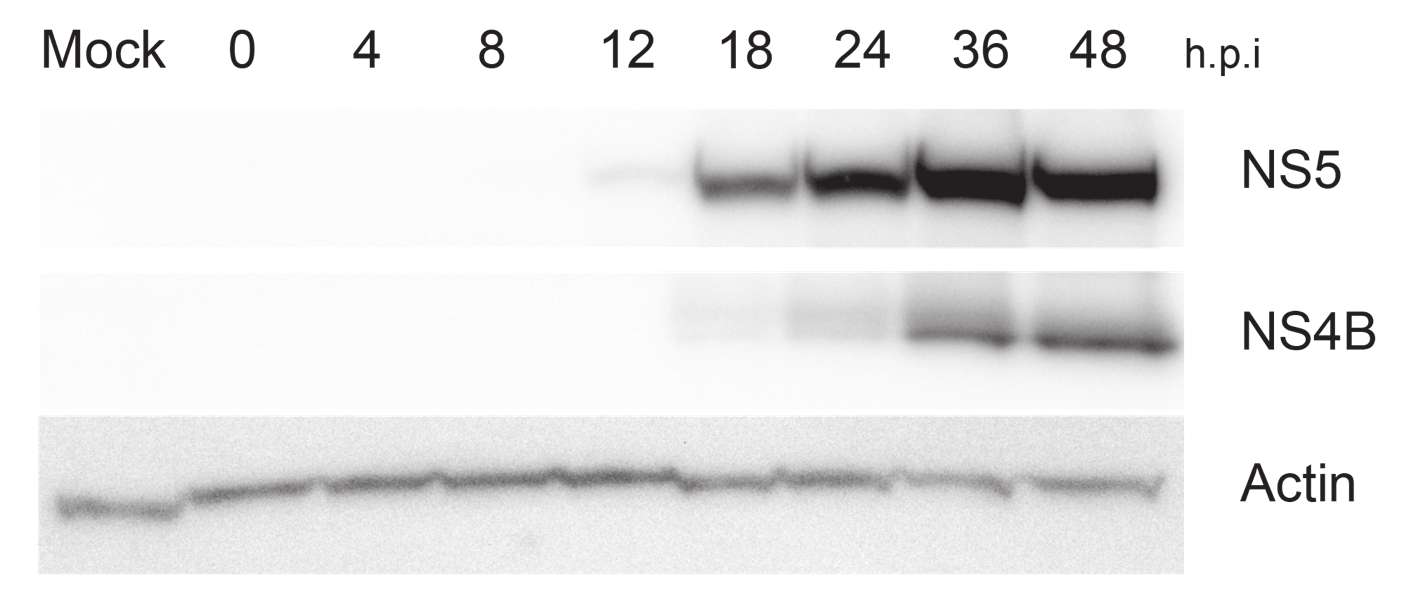


Vero cells were infected with ZIKV (strain PL Cal) at MOI of 3 and harvested at various time points denoted in the figure. Viral proteins were visualized using antibodies recognizing the corresponding proteins.
