# Supplementary material for: Discovery of a potent anti-Zika virus benzamide series targeting the viral protein NS4B
Source: PLoS Pathog. 2026 Apr 3;22(4):e1013609. doi: 10.1371/journal.ppat.1013609 (PMC13065080; doi:10.1371/journal.ppat.1013609)
Supplement: S4 Fig — (DOCX) [file ppat.1013609.s004.docx]

S4 Fig. Sequence alignment of the C-terminal region of ZIKV NS4B


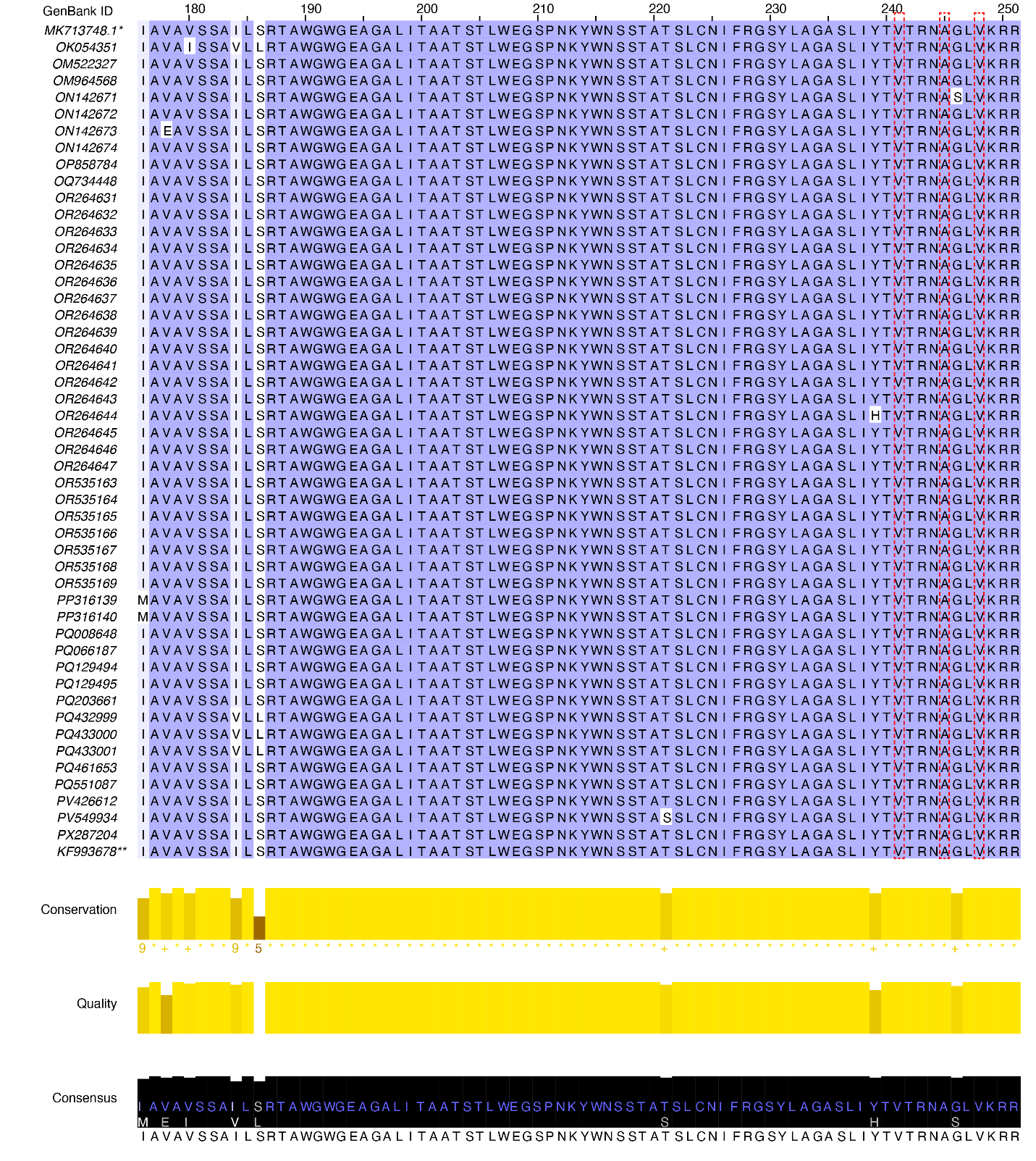


Sequence alignment was conducted with ClustalW using Jalview (version 2.11.5.1). * strain PRVABC59; **stain PLCal_ZV. Color scheme is based on the percent identity. The MWAC-3475 resistant mutations (i.e., V241, A245, and V248) were highlighted with red rectangles.
