# Supplementary material for: Discovery of a potent anti-Zika virus benzamide series targeting the viral protein NS4B
Source: PLoS Pathog. 2026 Apr 3;22(4):e1013609. doi: 10.1371/journal.ppat.1013609 (PMC13065080; doi:10.1371/journal.ppat.1013609)
Supplement: S5 Fig — (DOCX) [file ppat.1013609.s005.docx]

S5 Fig. Effect of MWAC-3475 on potential antiviral mechanisms.


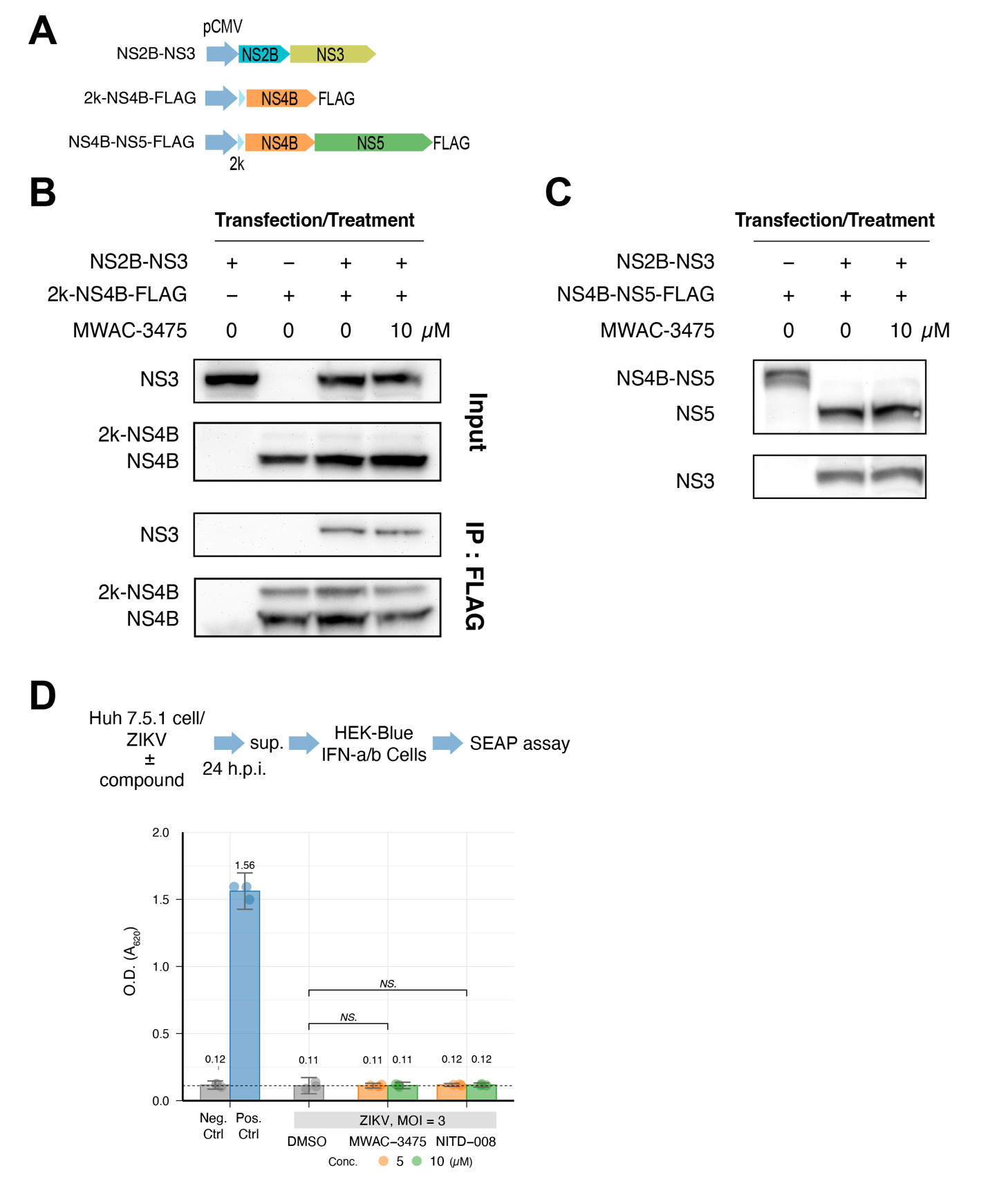


HEK-293T cells were transfected with plasmids using the PEI method, and transfected cells were treated with test compounds for 30 hours (A-C). (A) Schematic diagrams of NS protein expressing plasmids used in the experiment; pCDNA 3.1 was used as the plasmid backbone. (B) Co-immunoprecipitation of NS3 with NS4B. (C) Effect of MWAC-3475 on the NS4B-NS5 polyprotein processing. No changes resulting from MWAC-3475 were found. A representative image from more than 3 independent experiments. (D) Effect of MWAC-3475 on IFN induction after ZIKV infection. Huh 7.5.1 cells were infected with ZIKV (strain PL Cal) at MOI of 3 and the supernatants were harvested at 24 hours post infection. Induction of IFN a/b in the infected cells was measured by using HEK-Blue IFN-α/β cells as described in the Methods. NS: No significant in a one-way ANOVA with Dunnett’s post-hoc test.
