# Supplementary material for: Discovery of a potent anti-Zika virus benzamide series targeting the viral protein NS4B
Source: PLoS Pathog. 2026 Apr 3;22(4):e1013609. doi: 10.1371/journal.ppat.1013609 (PMC13065080; doi:10.1371/journal.ppat.1013609)
Supplement: S1 Text — (DOCX) [file ppat.1013609.s006.docx]

Supplementary Text

Chemistry protocols for the synthesis and characterization of non-commercial compounds that were evaluated.

General.

Materials were purchased from commercial vendors and used without purification. All moisture-sensitive reactions were performed under an argon atmosphere. Experiments were monitored by LCMS or TLC and visualized using an ultraviolet lamp (254 nm) or staining with KMnO_4_. Purification via silica gel flash column chromatography was performed using a Teledyne ISCO Combiflash® Rf+, Biotage® Isolera one, Biotage® Isolera four, Luknova silica gel cartridges, and Biotage® Sfar silica (60 µm). Biotage® Sfar C18 D (Duo 100 Å, 30 µm) cartridges were used with acetonitrile, water, and 0.1% TFA as solvents. All NMR data were collected at room temperature on a Brüker Ultrashield 400 MHz nuclear magnetic resonance spectrometer. Chemical shifts for ^1^H NMR spectra are reported in parts per million (ppm) relative to the residual solvent signal as an internal standard: DMSO (δ 2.50), CHCl_3_ (δ 7.26), acetone (δ 2.05), or MeOH (δ 3.31). Multiplicities are given as: s (singlet), d (doublet), t (triplet), q (quartet), m (multiplet), or br (broad). Coupling constants are reported as a J value in Hertz (Hz). Mass spectra were recorded on a Thermo Scientific 3000 LCQ Fleet system (ESI) using a Discovery® HS C18 HPLC column (10 cm x 2.1 mm, 5 µm) at 35 oC with UV detection at 254 nm. Flow rate was 0.7 mL/min using a solvent gradient of 5-95% B over 4 min (total run time = 6 min), where A = 0.1% formic acid in de-ionized H2O and B = 0.1% formic acid in ACN. Reverse-phase high-performance preparatory liquid chromatography was conducted using an Agilent 1290/1260 Infinity II HPLC system consisting of a G7114B VWD detector, a G7157A prep autosampler, two G7161A prep binary pumps, and a G7159B fraction collector. Data analysis was performed using Agilent’s ChemStation© software using an Agilent 10 Prep-C18 250 x 30.0 mm column with UV detection at 254 and/or 280 nm. The flow rate was 30 mL/min using a gradient of 10-80% B/A (A = 0.1% TFA in deionized H_2_O and B = 1:1 ACN/MeOH) for the necessary length of time to collect a pure sample.

***N-((exo)-bicyclo[2.2.1]heptan-2-yl)-4-(tert-butyl)benzamide*** (**MWAC-3989**).

 A mixture of 4-(tert-butyl)benzoic acid (50 mg, 0.28 mmol), *N*,*N*-Diisopropylethylamine (54 mg, 0.42 mmol), HATU (110 mg, 0.28 mmol), and racemic (exo)-bicyclo[2.2.1]heptan-2-amine (31 mg, 0.28 mmol) was stirred at room temperature for 3 h. After completion, the solvent was removed, and purification using prep-HPLC yielded pure product MWAC3989 (44.76 mg, 59% yield). ^1^H NMR (400 MHz, DMSO-*d*_6_) δ 7.98 (d, *J* = 6.8 Hz, 1H), 7.76 (d, *J* = 8.4 Hz, 2H), 7.45 (d, *J* = 8.5 Hz, 2H), 3.71 (q, *J* = 7.1 Hz, 1H), 2.23 (d, *J* = 4.1 Hz, 1H), 2.19 – 2.14 (m, 1H), 1.72 – 1.37 (m, 5H), 1.29 (s, 9H), 1.26 – 1.00 (m, 3H); MS(*m/z*): [M+H] calc’d for C_18_H_25_NO is 272.19, found 272.70.

***N*-((exo)-bicyclo[2.2.1]heptan-2-yl)-4-cyclopropylbenzamide** (**MWAC-3990**).

This compound was prepared according to the procedure for MWAC-3989 from 4-cyclopropylbenzoic acid, affording a pure product in 87% yield. ^1^H NMR (400 MHz, DMSO-*d*_6_) δ 7.95 (d, *J* = 6.7 Hz, 1H), 7.73 (d, *J* = 8.3 Hz, 2H), 7.12 (d, *J* = 8.3 Hz, 2H), 3.70 (q, *J* = 7.2 Hz, 1H), 2.28 – 2.18 (m, 1H), 2.21 – 2.13 (m, 1H), 1.96 (tt, *J* = 8.3, 5.0 Hz, 1H), 1.68 – 1.37 (m, 4H), 1.27 – 1.05 (m, 4H), 1.06 – 0.94 (m, 2H), 0.78 – 0.64 (m, 2H); MS(*m/z*): [M+H] calc’d for C_17_H_21_NO is 256.16, found 256.30.

***N*-((exo)-bicyclo[2.2.1]heptan-2-yl)-4-isopropylbenzamide** (**MWAC-3991**).

This compound was prepared according to the procedure for MWAC-3989 from 4-isopropylbenzoic acid, affording a pure product in 58% yield. ^1^H NMR (400 MHz, DMSO-*d*_6_) δ 7.98 (d, *J* = 6.8 Hz, 1H), 7.76 (d, *J* = 8.3 Hz, 2H), 7.30 (d, *J* = 8.3 Hz, 2H), 3.70 (q, *J* = 7.1 Hz, 1H), 2.93 (p, *J* = 6.9 Hz, 1H), 2.26 – 2.20 (m, 1H), 2.19 – 2.13 (m, 1H), 1.68 – 1.37 (m, 5H), 1.26 – 1.13 (m, 7H), 1.16 – 1.05 (m, 2H); MS(*m/z*): [M+H] calc’d for C_17_H_23_NO is 258.18, found 258.30.

***N-((endo)-bicyclo[2.2.1]heptan-2-yl)-4-(tert-butyl)benzamide*** (**MWAC-3992**).

This compound was prepared according to the procedure for MWAC-3989 but using the racemic endo amine starting material, affording a pure product in 37% yield. ^1^H NMR (400 MHz, DMSO-d6) δ 7.98 (d, J = 6.8 Hz, 1H), 7.88 (d, J = 8.6 Hz, 2H), 7.76 (d, J = 8.6 Hz, 2H), 3.70 (q, J = 7.1 Hz, 1H), 2.26 – 2.13 (m, 2H), 1.68 – 1.48 (m, 5H), 1.31 (s, 9H), 1.24 – 1.05 (m, 3H); MS(m/z): [M+H] calc’d for C_18_H_25_NO is 272.19, found 272.30.

***N*-((exo)-bicyclo[2.2.1]heptan-2-yl)-4-(2-hydroxypropan-2-yl)benzamide** (**MWAC-3995**).

This compound was prepared according to the procedure for MWAC-3989 from 4-(2-hydroxypropan-2-yl)benzoic acid, affording a pure product in 60% yield. ^1^H NMR (400 MHz, DMSO-*d*_6_) δ 7.98 (d, *J* = 6.7 Hz, 1H), 7.76 (d, *J* = 8.5 Hz, 2H), 7.51 (d, *J* = 8.4 Hz, 2H), 5.10 (s, 1H), 3.71 (q, *J* = 6.9, 2H), 2.28 – 2.18 (m, 1H), 2.21 – 2.13 (m, 1H), 1.70 – 1.38 (m, 11H), 1.31 – 1.03 (m, 4H); MS(*m/z*): [M+H] calc’d for C_17_H_23_NO_2_ is 274.17, found 274.30.

***N*-((exo)-bicyclo[2.2.1]heptan-2-yl)-4-ethylbenzamide** (**MWAC-3996**).

This compound was prepared according to the procedure for MWAC-3989 from 4-ethylbenzoic acid, affording a pure product in 55% yield. ^1^H NMR (400 MHz, DMSO-*d*_6_) δ 7.98 (d, *J* = 6.8 Hz, 1H), 7.76 (d, *J* = 8.2 Hz, 2H), 7.27 (d, *J* = 8.3 Hz, 2H), 3.71 (q, *J* = 7.0 Hz, 1H), 2.73 – 2.59 (m, 2H), 2.26 – 2.13 (m, 2H), 1.68 – 1.36 (m, 5H), 1.19 (td, *J* = 7.6, 5.3 Hz, 5H), 1.12 – 1.04 (m, 1H); MS(*m/z*): [M+H] calc’d for C_16_H_21_NO is 244.16, found 244.30.

***N*-((exo)-bicyclo[2.2.1]heptan-2-yl)-4-(trifluoromethyl)benzamide** (**MWAC-3997**).

This compound was prepared according to the procedure for MWAC-3989 from 4-(trifluoromethyl)benzoic acid, affording a pure product in 60% yield. ^1^H NMR (400 MHz, DMSO-*d*_6_) δ 8.33 (d, *J* = 6.6 Hz, 1H), 8.03 (d, *J* = 8.1 Hz, 2H), 7.83 (d, *J* = 8.3 Hz, 2H), 3.73 (q, *J* = 7.4 Hz, 1H), 2.27 – 2.17 (m, 2H), 1.71 – 1.38 (m, 5H), 1.26 – 1.07 (m, 3H); MS(*m/z*): [M+H] calc’d for C_15_H_16_F_3_NO is 284.12, found 284.27.

***N*-((exo)-bicyclo[2.2.1]heptan-2-yl)-4-(2-methoxypropan-2-yl)benzamide** (**MWAC-3998**).

This compound was prepared according to the procedure for MWAC-3989 from 4-(2-methoxypropan-2-yl)benzoic acid, affording a pure product in 54% yield. ^1^H NMR (400 MHz, DMSO-*d*_6_) δ 8.04 (d, *J* = 6.7 Hz, 1H), 7.80 (d, *J* = 8.4 Hz, 2H), 7.44 (d, *J* = 8.4 Hz, 2H), 3.71 (q, *J* = 7.1 Hz, 1H), 2.99 (s, 3H), 2.29 – 2.19 (m, 1H), 2.22 – 2.14 (m, 1H), 1.69 – 1.40 (m, 11H), 1.26 – 1.03 (m, 4H); MS(*m/z*): [M+H] calc’d for C_18_H_25_NO_2_ is 287.19, found 287.29.

**4-(tert-butyl)-N-cyclohexylbenzamide (MWAC-4000).**

This compound was prepared according to the procedure for MWAC-3989 but using cyclohexyl amine, affording a pure product in 62% yield. ^1^H NMR (400 MHz, DMSO-d6) δ 8.09 (d, J = 8.0 Hz, 1H), 7.77 (d, J = 8.5 Hz, 2H), 7.45 (d, J = 8.5 Hz, 2H), 3.75 (q, J = 7.4 Hz, 1H), 1.89 – 1.68 (m, 4H), 1.68 – 1.55 (m, 1H), 1.40 – 1.22 (m, 13H), 1.22 – 1.06 (m, 1H); MS(m/z): [M+H] calc’d for C_17_H_25_NO is 260.19, found 260.30.

**4-(tert-butyl)-N-cycloheptylbenzamide (MWAC-4001).**

This compound was prepared according to the procedure for MWAC-3989 but using cycloheptyl amine, affording a pure product in 62% yield. 1H NMR (400 MHz, DMSO-d6) δ 8.14 (d, J = 7.9 Hz, 1H), 7.76 (d, J = 8.5 Hz, 2H), 7.45 (d, J = 8.5 Hz, 2H), 3.94 (dtd, J = 12.3, 9.1, 4.5 Hz, 1H), 1.91 – 1.76 (m, 2H), 1.74 – 1.37 (m, 10H), 1.29 (s, 9H); MS(m/z): [M+H] calc’d for C_18_H_27_NO is 274.21, found 274.80.

***N*-((exo)-bicyclo[2.2.1]heptan-2-yl)-4-methylbenzamide** (**MWAC-4010**).

This compound was prepared according to the procedure for MWAC-3989 from 4-methylbenzoic acid, affording a pure product in 53% yield. ^1^H NMR (400 MHz, DMSO-*d*_6_) δ 7.98 (d, *J* = 6.7 Hz, 1H), 7.75 (d, *J* = 8.2 Hz, 2H), 7.24 (d, *J* = 8.1 Hz, 2H), 3.70 (q, *J* = 6.9, 1H), 2.35 (s, 3H), 2.27 – 2.13 (m, 2H), 1.68 – 1.37 (m, 5H), 1.25 – 1.05 (m, 3H); MS(*m/z*): [M+H] calc’d for C_15_H_19_NO is 230.15, found 230.50.

**(*S*)-4-(*tert-*butyl)-N-(1-(4-methoxyphenyl)ethyl)benzamide** (**MWAC-4168**).

A mixture of (*S*)-1-(4-methoxyphenyl)ethylamine (38 mg, 0.25 mmol), NaHCO_3_ (211 mg, 2.5 mmol), and 4-(*tert-*butyl)benzoyl chloride in (49 µL, 0.25 mmol) in DCM (2.5 mL) was stirred at room temperature overnight under argon. Upon completion, the solvent was removed and purification by silica gel chromatography afforded pure product MWAC-4168 (72 mg, 92% yield). ^1^H NMR (400 MHz, CDCl_3_) δ 7.70 (d, *J =* 8.0 Hz, 2H), 7.42 (d, *J =* 8.0 Hz, 2H), 7.32 (d, *J =* 8.0, Hz, 2H), 6.88 (d, *J =* 8.4 Hz, 2H), 6.26 (d, *J =* 6.8 Hz, 1H), 5.29 (quintet, *J =* 6.8 Hz, 1H), 3.80 (s, 3H), 1.59 (d, *J =* 6.8 Hz, 3H), 1.32 (s, 9H); MS(*m/z*): [M+H] calc’d for C_20_H_25_NO_2_ is 312.19, found 312.44.

**(*R*)-4-(*tert-*butyl)-N-(1-(4-methoxyphenyl)ethyl)benzamide** (**MWAC-4169**).

This compound was prepared according to the procedure for MWAC-4168 starting with (*R*)-1-(4-methoxyphenyl)ethylamine to afford pure product MWAC-4169 in 93% yield. ^1^H NMR (400 MHz, CDCl_3_) δ 7.70 (dd, *J =* 8.4, 1.6 Hz, 2H), 7.44 (dd, *J =* 8.4 Hz, 2H), 7.32 (dd, *J =* 8.4 Hz, 1.6 Hz, 2H), 6.89 (dd, *J =* 8.4 Hz, 1.6 Hz), 6.24 (d, *J =* 6.8 Hz, 1H), 5.30 (quintet, *J =* 6.8 Hz, 1H), 3.80 (s, 3H), 1.59 (d, *J =* 6.8 Hz, 3H), 1.33 (s, 9H); [M+H] calc’d for C_20_H_25_NO_2_ is 312.19, found 312.41.

**(*S*)-4-(*tert-*butyl)-N-(1-(3-methoxyphenyl)ethyl)benzamide** (**MWAC-4170**).

This compound was prepared according to the procedure for MWAC-4168 starting with (*S*)-1-(3-methoxyphenyl)ethylamine to afford pure product MWAC-4170 in 92% yield. ^1^H NMR (400 MHz, CDCl_3_) δ 7.64 (d, *J =* 8.0 Hz, 2H), 7.37 (d, *J =* 8.0 Hz, 2H), 7.22-7.18 (m, 1H), 6.91 (d, *J =* 7.6 Hz, 1H), 6.85 (s, 1H), 6.75 (dd, *J =* 8.4, 2.4 Hz, 1H), 6.24 (d, *J =* 7.6 Hz, 1H), 5.23 (quintet, *J =* 7.2 Hz, 1H), 3.73 (s, 3H), 1.52 (d, *J =* 7.2 Hz, 3H), 1.25 (s, 9H); [M+H] calc’d for C_20_H_25_NO_2_ is 312.19, found 312.54.

**(*S*)-4-(*tert-*butyl)-N-(1-(2-methoxyphenyl)ethyl)benzamide** (**MWAC-4164**).

This compound was prepared according to the procedure for MWAC-4168 starting with (*S*)-1-(2-methoxyphenyl)ethylamine to afford pure product MWAC-4164 in 55% yield. ^1^H NMR (400 MHz, CDCl_3_) δ 7.71 (d, *J =* 8.4 Hz, 2H), 7.44 (d, *J =* 8.8 Hz, 2H), 7.28-7.24 (m, 2H), 7.18 (d, *J =* 8.8 Hz, 1H), 6.94 (t, *J =* 7.2 Hz, 2H), 5.45 (quintet, *J =* 7.2 Hz, 1H), 3.93 (s, 3H), 1.55 (d, *J =* 7.2 Hz, 3H), 1.33 (s, 9H); [M+H] calc’d for C_20_H_25_NO_2_ is 312.19, found 312.60.

**(*S*)-4-(*tert-*butyl)-N-(1-(4-(hex-5-yn-1-yloxy)phenyl)ethyl)benzamide** (**MWAC-3986**, alkyne probe).

A mixture of (*S*)-4-(1-aminoethyl)phenol HCl (129 mg, 0.74 mmol), TEA (206 µL, 1.5 mmol), and 4-(*tert-*butyl)benzoyl chloride in (174 µL, 0.89 mmol) in DCM (7.4 mL) was stirred at room temperature overnight under argon. Upon completion, the solvent was removed and purification by silica gel chromatography afforded pure intermediate phenol product (*S*)-4-(*tert-*butyl)-N-(1-(4-hydroxyphenyl)ethyl)benzamide (124 mg, 56% yield). This compound (20 mg, 0.07 mmol), Cs_2_CO_3_ (24 mg, 0.07 mmol), and 6-iodohex-1-yne in (10 µL, 0.08 mmol) in DMF (700 µL) were together stirred at 50^o^C overnight in a sealed vial. Upon completion, the solvent was removed and purification by silica gel chromatography afforded pure product MWAC-3986 (16 mg, 63% yield). ^1^H NMR (400 MHz, CDCl_3_) δ 7.70 (d, *J =* 8.4 Hz, 2H), 7.43 (d, *J =* 8.4 Hz, 2H), 7.30 (d, *J =* 8.8 Hz, 2H), 6.88 (d, *J =* 8.8 Hz, 2H), 6.25 (d, *J =* 7.6 Hz, 1H), 5.29 (quintet, *J =* 6.8 Hz, 1H), 3.98 (t, *J =* 6.4 Hz, 2H), 2.27 (td, *J =* 7.2, 2.8 Hz, 2H), 1.97 (t, *J =* 2.6 Hz, 1H), 1.94-1.87 (m, 2H), 1.75-1.68 (m, 2H), 1.59 (d, *J =* 6.8 Hz, 3H), 1.32 (s, 9H); MS(*m/z*): [M+H] calc’d for C_25_H_31_NO_2_ is 378.24, found 378.35.

**FAM probe** (**MWAC-4163**).

A mixture of MWAC-3986 (8.3 mg, 0.02 mmol), fluorescein FAM azide 6-isomer (10 mg, 0.02 mg), sodium ascorbate (4 mg, 0.02 mmol), DIPEA (19 µL, 0.11 mmol), and CuI (2 mg, 0.01 mmol) in DMF (200 µL) was stirred at room temperature overnight in a sealed vial. Upon completion, purification by preparatory HPLC afforded pure product MWAC-4163. ^1^HNMR (400 MHz, CD_3_OD) δ 8.56 (d, *J =* 7.6 Hz, 1H), 8.10 (d, *J =* 8.0 Hz, 1H), 8.06 (d, *J =* 8.0 Hz, 1H), 7.75 (d, *J =* 8.0 Hz, 2H), 7.70 (s, 1H), 7.61 (s, 1H), 7.48 (d, *J =* 8.0 Hz, 2H), 7.28 (d, *J =* 8.4 Hz, 2H), 6.83 (d, *J =* 8.4 Hz, 2H), 6.68 (s, 2H), 6.59 (d, *J =* 8.8 Hz, 2H), 6.53 (d, *J =* 8.8 Hz, 2H), 5.17 (quintet, *J =* 6.8 Hz, 1H), 4.33 (t, *J =* 6.8 Hz, 2H), 3.93 (t, *J =* 5.2 Hz, 2H), 3.35-3.29 (m, 2H), 2.68 (t, *J =* 6.8 Hz, 2H), 2.09 (quintet, *J =* 6.8 Hz, 2H), 1.80-1.74 (m, 4H), 1.52 (d, *J =* 6.8 Hz, 3H), 1.32 (s, 9H); MS(*m/z*): [M+H] calc’d for C_49_H_49_N_5_O_8_ is 836.36 found 836.61.
