# Supplementary material for: Discovery of a potent anti-Zika virus benzamide series targeting the viral protein NS4B
Source: PLoS Pathog. 2026 Apr 3;22(4):e1013609. doi: 10.1371/journal.ppat.1013609 (PMC13065080; doi:10.1371/journal.ppat.1013609)
Supplement: S1 Table — (DOCX) [file ppat.1013609.s007.docx]

S1 Table

|  | ZIP | | HSA | | Bliss | | Loewe | | CSS |
| --- | --- | --- | --- | --- | --- | --- | --- | --- | --- |
| Combination | Mean | Max | Mean | Max | Mean | Max | Mean | Max |  |
| MWAC-3475 + NITD-008 | 5.47 | 42.08* | 7.15 | 48.08** | 5.29 | 37.35*** | 2.42 | 18.26* | 79.43 |

Synergy scores for MWAC-3475 and NITD-008

* A combination of 250 and 500 nM for MWAC-3475 and NITD-0008.

**500/1000, ***250/1000, ****500/125, †250/62.5 for MWAC-3475 and NITD-0008 (nM)
