# Supplementary material for: Discovery of a potent anti-Zika virus benzamide series targeting the viral protein NS4B
Source: PLoS Pathog. 2026 Apr 3;22(4):e1013609. doi: 10.1371/journal.ppat.1013609 (PMC13065080; doi:10.1371/journal.ppat.1013609)
Supplement: S2 Table — (DOCX) [file ppat.1013609.s008.docx]

S2 Table

| Molecular weight | 271 |
| --- | --- |
| Heavy atom count | 20 |
| Rotatable bonds | 2 |
| cLogP | 4.8 |
| cLogD (7.4) | 4.0 |
| # H-bond donors | 1 |
| # H-bond acceptors | 2 |
| CNS MPO score | 3.4 |
| tPSA | 29 |
| F(sp^3^) | 0.61 |
| CYP450 inhibition (1A2, 2C9, 2D6, 3A4) | < 50% at 10 µM for all |
| Kinetic aqueous solubility | < 0.4 mg/mL in PBS |
| Liver microsomal stability, Human | T_1/2_ = 56 min |
| Liver microsomal, Mouse | T_1/2_ = 5.9 min |
| Plasma protein binding | >97%, both human and mouse |

Physical properties and in vitro DMPK characteristics of MWAC-3508
