# Supplementary material for: Discovery of a potent anti-Zika virus benzamide series targeting the viral protein NS4B
Source: PLoS Pathog. 2026 Apr 3;22(4):e1013609. doi: 10.1371/journal.ppat.1013609 (PMC13065080; doi:10.1371/journal.ppat.1013609)
Supplement: S3 Table — (DOCX) [file ppat.1013609.s009.docx]

**S3 Table. Modification in the MWAC-3489 subseries for target ID**

| compound | MWAC ID | EC_50_-RLuc (µM) | EC_50_-CPE (µM) | CC_50_-Vero (µM) |
| --- | --- | --- | --- | --- |
|  | 3489 | 0.61 | 4.78 | >20 |
|  | 4168 | 0.23 | 0.95 | >20 |
|  | 4169 | 0.92 | >12.5 | 19.26 |
|  | 4170 | 1.24 | 8.88 | >20 |
|  | 4164 | 3.09 | >12.5 | 19.75 |
|  | 3986 | 0.21 | 0.74 | >50 |
