# Supplementary material for: Discovery of a potent anti-Zika virus benzamide series targeting the viral protein NS4B
Source: PLoS Pathog. 2026 Apr 3;22(4):e1013609. doi: 10.1371/journal.ppat.1013609 (PMC13065080; doi:10.1371/journal.ppat.1013609)
Supplement: S4 Table — (DOCX) [file ppat.1013609.s010.docx]

S4 Table. Peak titers of ZIKV-RLuc virus with resistant mutations

| Virus genotype | TCID50/mL |
| --- | --- |
| Wt | 3.56E+06 |
| A245T | 3.75E+05 |
| V248G | 4.15E+03 |
| V241L | 2.47E+05 |
