# Supplementary material for: Discovery of a potent anti-Zika virus benzamide series targeting the viral protein NS4B
Source: PLoS Pathog. 2026 Apr 3;22(4):e1013609. doi: 10.1371/journal.ppat.1013609 (PMC13065080; doi:10.1371/journal.ppat.1013609)
Supplement: S5 Table — (DOCX) [file ppat.1013609.s011.docx]

S5 Table. Primer sequences

| **Name** | **Sequences** | **Purpose** |
| --- | --- | --- |
| pmiRFP670_NS4B_Vec_FWD | GAAACGCTGGCTTGGTCAAGAGACGTTAGCTGAATTCTGCAGATATCCAGCACAGTG | pCDNA-miniRFPnano3 amplification |
| pmiRFP670_NS4B_Vec_REV | CTCTCCAACCATCCGAGTTCATTTGCGCTCTGCTGGATGGCGATGCCCATCA | pCDNA-miniRFPnano3 amplification |
| miniRFP_NS4B_Ins_FWD | TGATGGGCATCGCCATCCAGCAGAGCGCAAATGAACTCGGATGGTTGGAGAG | ZIKV NS4B amplification |
| miniRFP_NS4B_Ins_Rev | TGCTGGATATCTGCAGAATTCAGCTAACGTCTCTTGACCAAGCCAGCGTTTC | ZIKV NS4B amplification |
| pCDNA_miniRFP_ZNS4B REV | ATTGCCATTTGGTTGTCCTGGGGAGACATGGTACCAAGCTTAACTAGCCAGC | pCDNA-miniRFP-NS4B amplification |
| pCDNA_miniRFP_ZNS4B FWD | GCTTGATTACCGCCAATGAACTCGGAGCAAACCTGGACAAGATGCTGAACAC | pCDNA-miniRFP-NS4B amplification |
| 2K synthetic gene | GCTGGCTAGTTAAGCTTGGTACCATGTCTCCCCAGGACAACCAAATGGCAATCATCATCATGGTAGCAGTGGGTCTTCTGGGCTTGATTACCGCCAATGAACTCGGAGCAAACCTGGACAAGATGCTGAACAC | dsDNA for ZIKV 2K gene |
| ZIKV_NW_Fwd | CCGCTGCCCAACACAAG | Realtime PCR for ZIKV |
| ZIKV_NW_Rev | CCACTAACGTTCTTTTGCAGACAT | Realtime PCR for ZIKV |
| ZIKV_NW_probe | /5FAM/AGCCTACCTTGACAAGCA**A**TCAGACACTCAA /3IABKFQ/ | Realtime PCR for ZIKV |
| VERO_GADPH_Fwd | GGG TGT GAA CCA TGA GAA GTA T | Realtime PCR for Vero cell Gapdh |
| VERO_GADPH_Rev | GAG TCC TTC CAC GAT ACC AAA G | Realtime PCR for Vero cell Gapdh |
| VERO_GADPH_Probe | /5SUN/ACAACAGCC/ZEN/TCAAGATCGTCAGCA/3IABKFQ/ | Realtime PCR for Vero cell Gapdh |
